# Analysis of the cervical microbiome in women from the German national cervical cancer screening program

**DOI:** 10.1007/s00432-023-04599-0

**Published:** 2023-02-13

**Authors:** Mateja Condic, Claudio Neidhöfer, Damian J. Ralser, Nina Wetzig, Ralf Thiele, Martin Sieber, Lucia A. Otten, Leonie K. Warwas, Achim Hoerauf, Alexander Mustea, Marijo Parčina

**Affiliations:** 1 Department of Gynecology and Gynecological Oncology, University Hospital Bonn, Bonn, Germany; 2 Institute of Medical Microbiology, Immunology and Parasitology, University Hospital Bonn, Bonn, Germany; 3 Institute for Functional Gene Analytics, Bonn-Rhein-Sieg University of Applied Sciences, Sankt Augustin, Germany

**Keywords:** Cervicovaginal microbiome, Cervical cancer screening, HPV diagnostic, Colposcopy

## Abstract

**Purpose:**

Cervical cancer (CC) is caused by a persistent high-risk human papillomavirus (hrHPV) infection. The cervico-vaginal microbiome may influence the development of (pre)cancer lesions. Aim of the study was (i) to evaluate the new CC screening program in Germany for the detection of high-grade CC precursor lesions, and (ii) to elucidate the role of the cervico-vaginal microbiome and its potential impact on cervical dysplasia.

**Methods:**

The microbiome of 310 patients referred to colposcopy was determined by amplicon sequencing and correlated with clinicopathological parameters.

**Results:**

Most patients were referred for colposcopy due to a positive hrHPV result in two consecutive years combined with a normal PAP smear. In 2.1% of these cases, a CIN III lesion was detected. There was a significant positive association between the PAP stage and *Lactobacillus vaginalis* colonization and between the severity of CC precursor lesions and *Ureaplasma parvum*.

**Conclusion:**

In our cohort, the new cervical cancer screening program resulted in a low rate of additional CIN III detected. It is questionable whether these cases were only identified earlier with additional HPV testing before the appearance of cytological abnormalities, or the new screening program will truly increase the detection rate of CIN III in the long run. Colonization with *U. parvum* was associated with histological dysplastic lesions. Whether targeted therapy of this pathogen or optimization of the microbiome prevents dysplasia remains speculative.

**Supplementary Information:**

The online version contains supplementary material available at 10.1007/s00432-023-04599-0.

## Introduction

In recent years, the human microbiome has increasingly become the focus of scientific interest. The colonization of our body with microbiota is at least as diverse and complex as our somatic cell physiology (Sender et al. 2016). It is estimated that about 500–1000 different microorganisms simultaneously colonize our body (Turnbaugh et al. 2007). Alterations in the human microbiome, as well as interactions with the immune, endocrine, and nervous systems, have been linked to a variety of health changes and diseases, including cancer and their precursor lesions (Kostic et al. 2013; Helmink et al. 2019). The precise manner in which the microbiome influences the maintenance of health or the development of disease, however, is still far from being answered.

CC is predominantly caused by infection with human papillomavirus (HPV), in ≥ 99% with the high-risk (hr) HPV types 16 and 18 (Walboomers et al. 1999). About 90% of women are exposed to HPV infections during the course of their lives, and in only 10% of the cases the infection persists with a high risk of developing precancerous cervical intraepithelial lesions and CC (Shulzhenko et al. 2014). HPV persistence is co-induced by impaired immune reactions, and adverse accompanying effects exerted by the cervico-vaginal microbiome (Garrett 2015). There is growing scientific evidence for a relationship between a cervico-vaginal microbiome dominated by species other than lactobacilli, and a higher risk of HPV infection, HPV persistence and the development of CC and its precursor lesions (Mitra et al. 2015; Laniewski et al. 2020; Lin et al. 2020; Norenhag et al. 2020).

The development and improvement of molecular methods, in particular represented by bacterial 16S ribosomal RNA gene sequencing, has led to a deeper understanding of the cervico-vaginal microbiome (van de Wijgert et al. 2014). According to the presence of distinct bacterial species that are identified by 16S RNA sequencing, the cervico-vaginal microbiome is sometimes classified into five groups, designated as community state types (CST). In detail, CST I–III and CST V are characterized by an abundance of *Lactobacillus crispatus*, *L. gasseri*, *L. iners*, and *L. jensenii*, respectively, whereas, in contrast, CST IV shows a combination of diverse facultative anaerobes with low abundances of lactobacilli (Ravel et al. 2011). In reproductive-aged women, shifts from the *Lactobacillus*-dominated microenvironment are commonly observed during menses and sexual activity caused by a reduction of lactobacilli (Gajer et al. 2012). With increasing age and the decrease of estrogen and glycogen levels, *Lactobacillus* species are replaced by diverse anaerobes (Gliniewicz et al. 2019). This transformation of the cervico-vaginal site flora is associated with the genitourinary syndrome of menopause (Hummelen et al. 2011).

Dysbiosis of the lower female reproductive tract increases the risk for infections with STD (Martin et al. 1999). Further, the absence of Lactobacilli is associated with the increase risk of HIV and HSV transmission (Cherpes et al. 2003). Recent studies confirm that changes of the human microbiome can impair the symbiotic relationship between microorganisms and host, leading to the development of different cancer types and suggesting a role for microbiota in genesis of various malignancies (Bhatt et al. 2017; Lin et al. 2020; Norenhag et al. 2020).

For early detection of CC, an annual cytological examination program (PAP smear) has been introduced in Germany in 1971. Since then, incidence rates of CC dropped remarkably by 75% in the first decades but, however, incidence rates have stagnated in recent years. The PAP smear has a low sensitivity (60–80%), a false negative rate of 30% and false-positive rates ranging from 15 to 50% (Yim and Park 2007). Hence, in some European countries, a switch to primary HPV-DNA testing was established recently. HPV testing is a highly sensitive approach and the absence of hrHPV infection indicates a low risk for CC precursor lesions and CC development (Dillner et al. 2008). As part of the German National Cancer Plan, the Federal Joint Committee (G-BA) implemented an updated organized cervical cancer screening program starting in January 2020. Annual cytology screening remained unchanged for women between 20 and 34 years. For women of 35 years and older, a co-testing, comprising a Pap smear and an HPV test was introduced. In case of positive findings, women are referred for colposcopy (Bujan Rivera and Klug 2018). The aim of the present study was to evaluate the new screening program for the detection of high-grade precursor lesions and to investigate whether microbiome analyses could have a potential role in this screening.

## Methods

### Study design and population

The study cohort included women who were referred for colposcopy to the certified Colposcopy Centre at the Department of Gynecology and Gynecological Oncology of the University Hospital Bonn from November 2021 until February 2022. Colposcopy was indicated according to the guidelines of the new national cancer screening program (abnormal PAP smear finding and/or a positive result for hrHPV).

Routine colposcopy was performed including the application of acetic acid. In cases of TZ type 1 or TZ type 2 (Quaas et al. 2013), a targeted biopsy was performed from the most conspicuous lesion. In case of a TZ type 3 with no visible lesion on the ecto-cervix, an endo-cervical curettage was performed.

Clinical data regarding nicotine abuse, menopause status, HPV vaccination, the application of local suppositories, the intake of hormonal contraceptives or hormone replacement therapy, the presence of an intrauterine device (IUD-copper or hormonal) and the last sexual intercourse were obtained from patient questionnaires and the clinical database.

### Histopathological analysis and HPV diagnostics

The taken biopsies were histopathologically classified into benign, low squamous intraepithelial lesions (LSIL/CIN I), and high squamous intraepithelial lesions (HSIL) according to the 2014 WHO classification. HSIL lesions were further sub-classified into CIN II and CIN III lesions according to Richart (Richart 1973).

In women above 35 years, HPV status was available as a part of the new cancer screening program. Due to the use of different HPV molecular detection assays and, therefore, inconsistent data for specific HPV types, analyses with respect to HPV were limited to low risk (lr) and hrHPV. In the presence of hrHPV, it was differentiated whether hrHPV types 16 and/or 18 were present.

HPV diagnostics were repeated from all samples with the Anyplex II HPV28 Detection (Seoul, South Korea) that detects 19 hrHPV: 16, 18, 26, 31, 33, 35, 39, 45,51, 52, 53, 56, 58, 59, 66, 68, 69, 73, 82; and nine low carcinogenic risk HPV types: 6, 11, 40, 42, 43, 44, 54, 61, 70. It was performed strictly following the manufacturer’s instructions. DNA was extracted on a Seegene NIMBUS (Seoul, South Korea) and analyzed on a CFX96 real-time PCR instrument (Bio-Rad Laboratories, Inc., Hercules, California, USA). Data recording and analysis were automated using the Seegene Viewer software.

Women, in which no biopsy was taken for histological analysis and women with pathologies of the vulva were excluded from the study.

### Sample collection and preparation for sequencing

During the colposcopic examination, before the application of acetic acid, a flocked swab (eNAT® system, Copan Italia, Brescia, Italy) was taken by three experienced gynecologists from the cervical canal. The swabs were stored at 4 °C and subsequently processed within 2–9 days.

Highly purified DNA was extracted from all samples using the column-based ZymoBIOMICS DNA Miniprep Kit (Zymo Research Europe GmbH, Freiburg, Germany). The isolation was performed strictly according to the manufacturer’s instructions. The crucial mechanical lysis step of the samples was performed by Precellys^®^ Evolution homogenizer from Bertin Technologies SAS (Bretonneux, France). At the end of the extraction process, the DNA was eluted to 100 uL volume and qualitatively and quantitatively evaluated using the NanoDrop OneC, Thermo Fisher Scientific Inc. (Waltham, MA, USA).

16S rRNA gene sequencing libraries were constructed from each sample using the Quick-16S NGS Library Prep Kit (Zymo Research Europe GmbH, Freiburg, Germany) with its included V1–V2 primer pairs. Each run included 94 samples, the positive control included in the kit, and a negative control. For quantitative PCR, quality control, and normalization purposes, the Bio-Rad CFX96 Real-Time PCR Detection System (Bio-Rad Laboratories, Inc., Hercules, California, USA) was utilized.

After pooling, the DNA was quantified with the QuantiFluor^®^ dsDNA System on the Quantus^™^ Fluorometer (both: Promega GmbH, Walldorf, Germany) and diluted strictly according to the Illumina-protocol for MiSeq sample preparation. For the final library, a loading concentration of 10 pm was chosen and a 10% Illumina v3 PhiX spike-in control was added before running it on the Illumina MiSeq platform with a 500cycle v2 Illumina MiSeq Reagent Kit (all three: Illumina, San Diego, CA, USA).

### Bioinformatic analysis

The bioinformatic analysis included three main parts, starting with the preprocessing of raw paired end reads. Following the preprocessing, the sequences were assigned to taxonomies. Finally, a statistical and graphical evaluation was performed on the resulting taxa.

QIIME2 (Bolyen et al. 2019) was used for both preprocessing and classification of the data. With the plugin tool DADA2 (Callahan et al. 2016), forward and reverse reads were trimmed from the 3’ end at position 249, while shorter reads as well as low-quality reads got discarded. DADA2 was also used to perform error correction, merging of forward and reverse reads if there was an overlap of at least 12 base pairs, and chimera removal.

The processed sequences were clustered into OTUs (operational taxonomic units) of 100% sequence identity and assigned to taxa, using a classifier trained on full-length sequences of SILVA (Quast et al. 2013). The trained classifier was provided by QIIME2 using scikit-learn 0.24.1 and the plugin tool q2-feature-classifier (Bokulich et al. 2018; Robeson et al. 2021).

### Statistical analysis

Statistical analysis was performed using Stata version 14 for the clinical data and Datatab version 1.12.1 for taxa frequency comparisons and correlation with clinical parameters. P values less than 0.05 were considered statistically significant.

### Ethics statement

The study was approved by the Ethics Committee of the Medical Faculty of the University of Bonn (vote: 128/21). All methods were carried out in accordance with relevant guidelines and regulations. Informed consent was obtained from all subjects.

## Results

### Participant characteristics and clinical results

The study cohort included 310 women. All relevant clinicopathological parameters are summarized in Table 1. The mean age of the study cohort was 44.6 years (± standard deviation (SD) 12.4 years). 72.9% of the women were premenopausal, and 27.1% were postmenopausal. 30.3% of the patients were active smokers. Within the whole cohort, 12.3% of the patients had been vaccinated against HPV. Among the subgroup of women ≤ 30 years, 62.2% had been vaccinated against HPV. In the subgroup of premenopausal women, 29.2% used hormonal contraceptives, and 13.3% had an IUD. Of these, 26.7% had a copper, 63.3% a Mirena ^®^ IUD, 6.7% a Jaydess ^®^, and 3.3% a Kyleena ^®^ IUD. In the subgroup of postmenopausal women, 8.3% received hormone replacement therapy. The proportion of postmenopausal women who used vaginal suppositories (estriol) was 21.4%. Our analysis included five pregnant women.

**Table 1:** Clinicopathological characteristics of the entire cohort

Clinicopathological parameter	
Age (years)	
Mean (± SD)	44.6 ± 12.4
Min–max	20–82
Menopausal status	
Pre	226 (72.9%)
Post	84 (27.1%)
Smoker	
No	216 (69.7%)
Yes	94 (30.3%)
HPV vaccination	
No	272 (87.7%)
Yes	38 (12.3%)
HPV vaccination < 30 years	
No	14 (37.8%)
Yes	23 (62.2%)
Hormonal contraceptives (premenopausal)	
No	160 (70.8%)
Yes	66 (29.2%)
Intrauterine device (IUD) (premenopausal)	
No	196 (86.7%)
Yes	30 (13.3%)
IUD	
Cooper	8 (26.7%)
Hormonal	22 (73.3%)
Hormonal replacement therapy (postmenopausal)	
No	77 (91.7%)
Yes	7 (8.3%)
Vaginal suppositories (postmenopausal)	
No	66 (78.6%)
Yes	18 (21.4%)
HPV high-risk status	
Negative	31 (10.0%)
Positive	262 (84.5%)
Unknown	17 (5.5%)
HPV 16/18	
Negative	183 (69.8%)
Positive	79 (30.2%)
Clinicopathological parameter	
Pap smear cytology	
I/IIa	162 (52.3%)
IIp	40 (12.9%)
IIg	6 (1.9%)
IIIp	11 (3.5%)
IIIg	4 (1.3%)
IIID1	46 (14.8%)
IIID2	22 (7.1%)
IVa-p	18 (5.8%)
IVa-g	1 (0.3%)
Histological diagnosis	
No CIN	201 (64.8%)
LSIL CIN I	51 (16.5%)
HSIL CIN II	36 (116%)
HSIL CIN III	22 (7.1%)
Histological diagnosis, Pap I/IIa, HPV high-risk pos	
No CIN	112 (77.8%)
LSIL CIN I	20 (139%)
HSIL CIN II	9 (6.3%)
HSIL CIN III	3 (2.1%)
Surgical therapy	
No	263 (84.8%)
Yes	47 (15.2%)
Type of surgical therapy	
LEEP conization	38 (80.9%)
Hysteroscopy/curettage	3 (6.4%)
Hysterectomy	2 (4.3%)
Laser vaporization	4 (8.5%)
Therapy of CIN III	
LEEP conization	19 (86.4%)
Hysterectomy	1 (4.5%)
No surgical therapy	2 (9.1%)
Therapy of CIN II	
LEEP conization	19 (52.8%)
Hysterectomy	1 (2.8%)
No surgical therapy	16 (44.4%)

84.5% of the whole study cohort were positive for a hrHPV type. The most prevalent subtypes were HPV types 16 und 18 in 30.2% of the cases. In 5.5% of the study cohort, HPV status was not available and 10.0% were negative for hrHPV types.

Most interestingly, 52.3% of the study cohort had a regular Pap smear (I or IIa, according to the Munich III classification). They were referred to a colposcopy due to a positive status for hrHPV in two consecutive years. This approach corresponds to the new guidelines. Among the subgroup with a positive hrHPV status and normal cytology, 8.4% of the women had an HSIL. A CIN III was detected in only 2.1% (3/144).

In the entire study cohort, histological examination revealed in 16.5% of the cases a CIN I, in 11.6% a CIN II, and in 7.1% a CIN III. 15.2% of all patients received surgical therapy due to precursor lesions of the cervix, with 80.9% receiving a LEEP conization, 6.4% a hysteroscopy with curettage, 4.3% a hysterectomy, and 8.5% laser vaporization. Among the 22 patients diagnosed with a CIN III lesion, 19 received conization and one a hysterectomy. Two patients with CIN III did not receive surgical treatment: one woman was pregnant at the time of diagnosis, and one was 20 years old. In this case, a close surveillance every three months was scheduled, which is in line with guidelines. Among the CIN II subgroup, 52.8% received a LEEP conization and one woman a hysterectomy. In 44.4% of the cases, a follow-up was scheduled in 6 months according to the guidelines. In cases with LSIL/CIN I, no surgical therapy was performed, and a follow-up colposcopy in 6 months was scheduled.

### HPV Diagnostic

HPV diagnostic was repeated in all patients. In 252 cases, HPV status determined within the CC screening program was in concordance with our analysis. In 18 cases, the comparison was not possible as these patients did not receive prior HPV testing or our analysis was invalid. In 40 cases, HPV HR diagnostic showed a discrepancy in the results: In 20 cases, that were initially tested negative for HPV HR within the CC screening program, a positive hrHPV status was determined in our analysis. Among these cases, 11 were positive for HPV 16. The medical history showed that 9 patients had cervical dysplasia before. In the actual biopsy, none of the patients had an HSIL. In 20 cases that were initially hrHPV-positive, no hrHPV infection was detected in our analysis. Five of these patients were initially positive for HPV 16/18. As observed for the counterpart subgroup, none of the patients had an HSIL.

In 17 cases, HPV diagnostic was not performed previously, as patients were younger than 35 years. These patients were referred for colposcopy due to an abnormal PAP smear. We found a positive HPV HR status in 16 patients; histology showed in 5 cases an LSIL and in 7 cases an HSIL.

### Cervical microbiome profiles

The 310 sequenced cervical samples generated a total of 31,881,480 reads with a mean read count of 102,843 per sample. Of these samples, 293 passed the minimum quality filter (> 1500 reads and > 1000 merged reads).

**Fig. 1:**
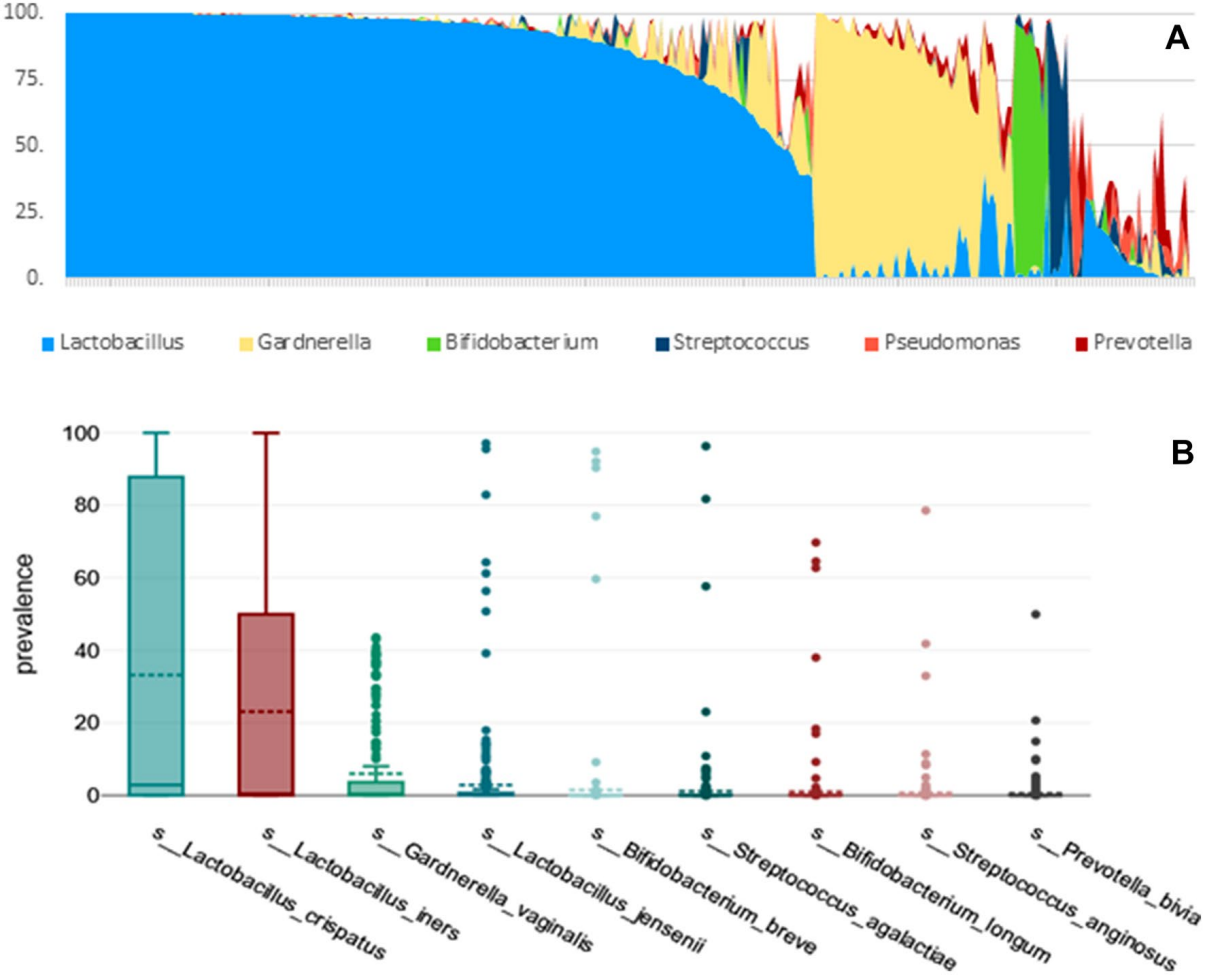
Genus-level cervical microbiota profiles **A**. Prevalence of species with an average prevalence > 0.5% **B**

Cervical microbiota profiles were classified into 5 groups based upon the dominant (or at least > 30% relative abundance) taxa observed within each sample at the genus level (see Fig. 1). Accordingly, 194 microbiomes were predominated by *Lactobacillus* (66.21%), 52 by *Gardnerella* (17.75%), 9 by *Bifidobacterium*, 6 by *Streptococcus*, 2 by *Pseudomonas*, 1 by *Prevotella* and in 29 cases (9.9%) none of these genera predominated.

Nine bacterial species had an average prevalence of > 0,5%, namely *Lactobacillus crispatus* (mean prevalence 33,21%)*, Lactobacillus iners* (23,12%)*, Gardnerella vaginalis* (5,98%)*, Lactobacillus jensenii* (2,84%)*, Bifidobacterium breve* (1,47%)*, Streptococcus agalactiae* (1,13%)*, Bifidobacterium longum* (1,01%),* Streptococcus anginosus* (0.72%), and *Prevotella bivia* (0.58%) (Fig. 1B).

The relative abundance of *L. crispatus* was negatively correlated with *L. iners* (*r* =  − 0.41, *p* < 0.001), *G. vaginalis* (*r* =  − 0.36, *p* < 0.001), *L. jensenii* (*r* =  − 0.12, p = 0.043), and *P. bivia* (*r* =  − 0.13, *p* = 0.028). The relative abundance of *L. iners* was negatively correlated with *L. crispatus and G. vaginalis* (*r* =  − 0.21, *p* < 0.001). No other significant correlations between these species were observed.

### Correlations between clinical and demographic variables and the microbiome

**Fig. 2:**
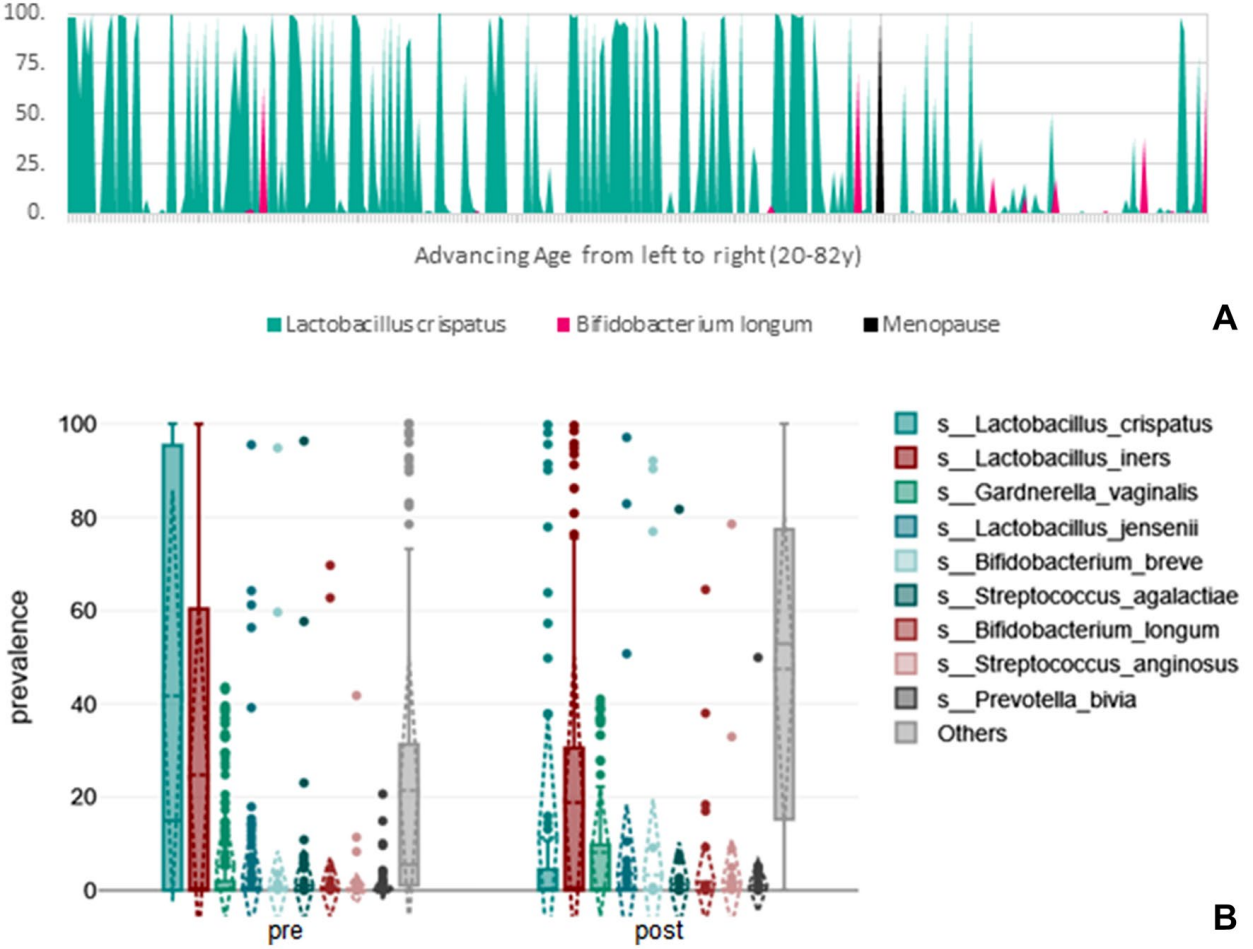
Visualization of the influence of menopause and age on *L. crispatus* and *B. longum*, respectively (A), and the displacement of *L. crispatus* by other species **B**

The patient’s age was correlated negatively with *L. crispatus* (*r* =  − 0.3, *p* < 0.001) and positively with *G. vaginalis* (*r* = 0.15, *p* = 0.011) and *B. longum* (*r* = 0.14, *p* < 0.016). However, performing multiple linear regression analysis to examine the influence of the menopausal state, revealed that only the *B. longum* was associated with age (*p* <  = 0.027). As depicted in Fig. 2, being postmenopausal correlated significantly negatively with *L. crispatus* (rpb = − 0.32, *n* = 293, *p* =  < 0.001) and positively with the genus *Pseudomonas* (rpb = 0.2, *p* = 0.001), *Prevotella* (rpb = 0.15, *p* = 0.011), *Cutibacterium* (rpb = 0.14, *p* = 0.015), *Atobium* (rpb = 0.13, *p* = 0.027), *Staphylococcus* (rpb = 0.14, *p* = 0.014), *Dialister* (rpb = 0.16, *p* = 0.008), *Acinetobacter* (rpb = 0.14, *p* = 0.017), *Oscillospirales* (rpb = 0.19, *p* = 0.001), and *Fusobacterium* (rpb = 0.15, *p* = 0.008) (*n* = 293 for all). The cervical microbiomes of postmenopausal patients displayed higher richness (t(124.46) =  − 2.71, *p* = 0.008, 95% confidence interval [− 40.29, − 6.23]) and higher fisher-alpha diversity (t(116.25) =  − 3.13, *p* = 0.002, 95% confidence interval [− 8.11, − 1.82]).

Premenopausal women with an IUD displayed lower richness in their cervical microbiome (t(45.87) = 2.27, *p* = 0.028, 95% confidence interval [2.19, 36.46]) than those without. However, one-factor analysis of variance showed that there was no significant difference between not having an IUD, having a hormonal IUD, or a copper IUD and the variable richness *F* = 1.84, *p* = 0.161 (Fig. A1 in the Appendix displays differences among IUDs that were not statistically significant). Among premenopausal women, intake of oral contraceptives was linked to a higher prevalence of *L. crispatus* (t(209) = − 3.42, *p* = 0.001, 95% confidence interval [− 35.48, − 9.43]) and a lower prevalence of *L. iners* t(182.96) = 4.45, *p* =  < 0.001, 95% confidence interval [10.58, 27.58]. Among postmenopausal women taking hormone replacement therapy, no such differences were observed (Fig. A2 in the Appendix displays these differences that were not statistically significant). Smoking was positively correlated with the genus *Veillonella* (rpb = 0.16, *n* = 293, *p* = 0.008).

### Correlations between cytology/histology and the microbiome

The result of the Pearson correlation showed that there was a significant low positive association between Pap stage and the order *Lactobacillales* (r(291) = 0.15, *p* = 0.008), the genus *Lactobacillus* (r(291) = 0.15, *p* = 0.008), the genus *Bacillus* (r(291) = 0.21, *p* =  < 0.001) and with *Lactobacillus vaginalis* (r(291) = 0.22, *p* =  < 0.001). When excluding postmenopausal patients from the analysis, only the positive associations with the genus *Bacillus* (r(209) = 0.24, *p* =  < 0.001) and with *Lactobacillus vaginalis* (r(209) = 0.24, *p* =  < 0.001) remained significant.

**Fig. 3:**
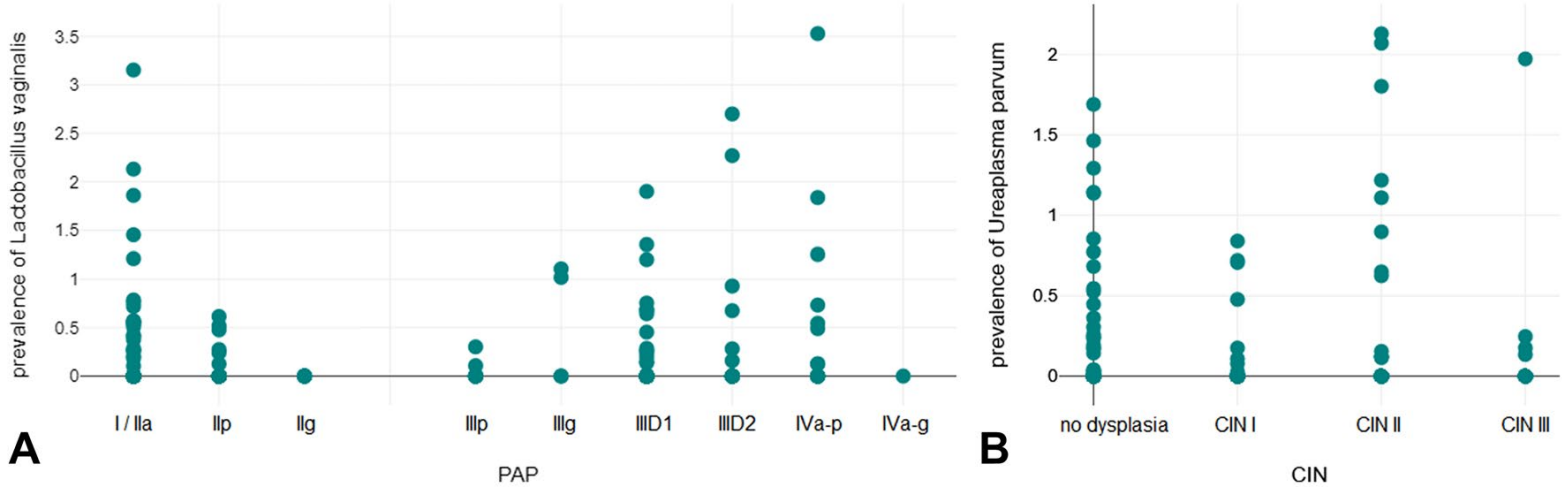
Scatter diagram on the prevalence of *L. vaginalis* by cytological stage (**A**) and *U. parvum* by histological stage (**B**)

Further, there was a significant low positive association between histological stage and the genus *Lactobacillus* (r(291) = 0.12, *p* = 0.038), the genus *Bacillus* (r(291) = 0.17, *p* = 0.004),and *L. vaginalis* (r(291) = 0.16, *p* = 0.006). In addition, there was a low, positive correlation between histological stage and *Sneathia sanguinegens* (r(291) = 0.14, *p* = 0.02), the order *Mycoplasmatales* (r(291) = 0.19, *p* = 0.001), the genus *Ureaplasma* (r(291) = 0.18, *p* = 0.002), and *Ureaplasma parvum* (r(291) = 0.17, *p* = 0.004). Multiple linear regression analysis to examine the influence of age, menopausal state, smoking, and IUD in addition to Pap stage and histology, revealed that Pap stage remained associated with the genus *Bacillus* (*p* = 0.033), and *L. vaginalis* (*p* = 0.021). Histological stage remained associated with the order *Mycoplasmatales* (*p* = 0.015), the genus *Ureaplasma* (*p* = 0.016), and *U. parvum* (*p* = 0.15) (see Fig. 3).

## Discussion

This study was conducted to evaluate the newly implemented German national CC screening program combined with an analysis of the cervical microbiome in patients referred to colposcopy according to the new guidelines.

There is broad scientific evidence from randomized controlled trials and meta-analyses that screening for HPV is more sensitive in the detection of cervical intraepithelial neoplasia grade III (CIN III) and CC than conventional cervical cytology (Naucler et al. 2007; Anttila et al. 2010; Ronco et al. 2010; Rijkaart et al. 2012). Integration of HPV testing into CC screening programs led to a decrease in CC incidence (Kjaer et al. 2010). This is, in particular, attributable to higher detection rates of cervical adenocarcinoma and its precursor lesions, as this subgroup is often underdiagnosed by cytological methods (Castle et al. 2010; Katki et al. 2011). Trials showed that women that are negative for hrHPV display a very low risk for the development of CIN III CC precursor lesions or CC (Dillner et al. 2008; Mesher et al. 2010). Based on these data, the new national CC screening program was set up in Germany with the implementation of a Pap smear/HPV co-testing for women aged 35 years and older.

Our study showed that 52.3% of the patients were referred for colposcopy due to an hrHPV-positive result in two consecutive years and normal cytology in both years. Among this subgroup, histological examination revealed only 3 cases of CIN III (2.1%) and 9 cases of CIN II (6.3%). Data from other studies reported a CIN III incidence range of 3–7% in women with normal cytology and a positive high-risk HPV test (Petry et al. 2003; Thrall et al. 2010). Another study from Germany that evaluated co-testing in women older than 30 years showed CIN III lesions in 9.2% of hrHPV-positive/ normal-cytologic cases (Luyten et al. 2014). The implementation of colposcopy for Pap-normal/hrHPV-positive women in two consecutive years had the goal of diagnosing approximately 10% CIN III detected lesions. One finding is that the new screening program leads to an increased need for colposcopies and histological examinations performed. In our study, the supplementary HPV testing identified 3 cases of CIN III that would not have received histological assessment in the old screening program. Whether the number of CIN III remains the same in the long term, and additional HPV testing only detects them earlier before cytological abnormalities are detectable, must be clarified in further studies.

In 17 cases, the HPV status was not available as women were younger than 35 years and referred for colposcopy due to an abnormal Pap smear. Histological examination revealed in 5 cases a LSIL and 7 cases an HSIL lesion. HPV analyses showed a positive result for hrHPV in 16/17 cases. As all precursor lesions among these patients were detected by Pap smear, there was no additional benefit of HPV testing. However, this must be interpreted with caution as women < 35 with unremarkable PAP smears but positive hrHPV status were not included in the study and are not referred for a colposcopy within the current screening algorithm.

In our study, we repeated the HPV diagnostic with a test based on the Anyplex II HPV28 Detection for all enrolled patients. In 40 cases, there were deviating results from the initial testing for hrHPV. In 20 cases, that initially tested positive, the new negative result can be explained by spontaneous regression, as the time difference between the two analyses was 3–5 months. In 20 initial hrHPV negative cases, we found a positive result for hrHPV, with even 11 cases being positive for HPV 16. Divergence of these results can be explained by different sensitivity of HPV tests, a new infection with hrHPV, or a reactivation of a hrHPV infection in the meantime. A crucial step in an HPV-based cervical cancer screening program is the selection of an appropriate HPV test (Arbyn et al. 2015). As HPV infections are very common, with a high tendency for spontaneous regression, the positive predictive value for all HPV tests is relatively low. On the global market, 82% of the HPV tests lack any published analytical and/or clinical evaluation (Poljak et al. 2016). In the case of this study, Anyplex II HPV28 Detection was chosen as the broadest CE/IVD PCR assay, and extensively validated in the Vigilant Framework settings (Bonde et al. 2018).

Most of the available HPV tests are DNA-based and can only discriminate between the presence and absence of HPV-specific DNA. Hence, these tests are not able to discriminate between an active or inactive infection (Benevolo et al. 2011). Tests that use E6/E7 mRNA detection demonstrated higher clinical specificities than DNA-based tests, as E6/E7 mRNA is only found in actively infected cells (Ratnam et al. 2010; Arbyn et al. 2013). Currently, there are a variety of approved HPV tests available in Germany for screening, both DNA- and mRNA-based, with most using DNA test kits. Caution is needed when interpreting HPV results, as there are many different assays and positive HPV-DNA does not necessarily mean that an active infection is present. Future studies evaluating the new cancer screening program will need to clarify whether supplemental HPV testing improves the detection rate of CIN III in the long term and not just increases the number of examinations performed (colposcopies and histologic assessments). In future, more emphasis should be given to the selection of HPV assays, as the conclusions from mRNA and DNA assays differ significantly.

The production of lactic acid leading to a pH below 4,5 and antimicrobial substances such as bacteriocins, the competition for nutrients to counteract the overgrowth by other microorganisms, and the modulation of the local immune response are the main mechanisms of the protective role of lactobacilli (Aroutcheva et al. 2001). High estrogen levels and especially the glycogen content of the vaginal epithelium (Mirmonsef et al. 2016) lead to an environment dominated primarily by *L. crispatus*,* L. gasseri*, and *L. jensenii *(Gajer et al. 2012). The production of lactic acid is one central mechanism by which microorganisms protect themselves from viruses and competitors (Mitra et al. 2016). Further, *L. iners* dominance over *L. crispatus* has been associated with a higher risk for intraepithelial squamous lesions and cancer (Norenhag et al. 2020). A study analyzing the microbiota of HPV-positive and negative women demonstrated that *L. gasseri* is associated with a higher HPV elimination (Brotman et al. 2014). Accordingly, CST IV is associated with cervical abnormalities, low-grade squamous intraepithelial lesions (LSIL), high-grade SIL (HSIL), and cervical cancer. Comparing LSIL and HSIL samples, there was a microbiome shift to a greater abundance of *Sneathia sanguinegens*,* Anaerococcus tetradius*, and *Peptostreptococcus anaerobius* and a lower abundance of *L. jensenii* with HSIL (Mitra et al. 2015). These data suggest a major role of the vaginal and cervical microbiome in the development of precancerous lesions of the cervix. Nevertheless, the most important previously published papers on the role of the cervical microbiome in the development of cervical carcinoma were limited to 169 (Mitra et al. 2015), 137 (Zhang et al. 2018), 126 (Seo et al. 2016), 120 (Oh et al. 2015), 94 (Wu et al. 2021), 92 (Tango et al. 2020), 47 (Kwon et al. 2019) and 32 (Audirac-Chalifour et al. 2016) patients, respectively. While despite all limitations in comparing results of microbiome studies, the relative patterns in various conditions would be expected to be reasonably consistent (Berman et al. 2020). This does not necessarily hold true if different primer pairs targeting 16 s-rDNA are used. On the one hand, universal V3/V4 Primer pairs do, for example, allow for better vaginal community state types assignment than universal V1/V2-based primers, detect more taxa, and generally present a higher abundance of *Gardnerella vaginalis *(Graspeuntner et al. 2018). In silico, on the other hand, V1/V3 primers seemed to perform at least as good as V3/V4 (Hugerth et al. 2020), and optimized V1/V2 primers, such as those used in our study, cover *Bifidobacteria* and disease-associated taxa, such as *G. vaginalis* and *Chlamydia trachomatis *(Frank et al. 2008; Zhang et al. 2019), while better differentiating among *Lactobacilli *(Zhang et al. 2019). In our study, we not only see more *G. vaginalis* than would be expected with universal V1/V2 primers, but also similar proportions of *Lactobacillus*-, *Gardnerella*-, and mixed-flora-dominated microbiomes to those observed in the largest shotgun metagenomics study performed to date of the cervical microbiome (Jie et al. 2021).

Primers previously used included, most importantly, universal V1/V2 (Mitra et al. 2015), V1/V3 (Oh et al. 2015; Seo et al. 2016), V3/V4 (Audirac-Chalifour et al. 2016; Zhang et al. 2018), and such targeting the V4 region (Wu et al. 2021). Nevertheless, even among studies using the same primer pairs, results substantially differed. While some argued that anaerobes, greater alpha diversity, and consequently lower levels of *Lactobacilli* seemed associated with a bad prognosis (Mitra et al. 2015; Audirac-Chalifour et al. 2016; Wu et al. 2021), others did not find differences linked to *Lactobacilli* or associations with anaerobes found in earlier studies (Oh et al. 2015; Seo et al. 2016; Zhang et al. 2018). Both studies targeting the V1/V3 region found excessively large proportions of *Fannyhessea (Atopobium) vaginae* and, respectively, little *G. vaginalis.* All studies agreed, however, that more studies involving larger sample sizes are needed, given the possible bias occurring with smaller sample sizes.

We are skeptical of differences linked to rare taxa in previous studies, also due to partially small study populations. Despite the V3/V4 primers being described as detecting more taxa (Graspeuntner et al. 2018), meta-transcriptome analyses have shown that only a couple of dominant genera contribute to most of the bacterial transcripts (Arroyo Muhr et al. 2021).

Rather than with neoplasia, we see the most remarkable differences in terms of alpha diversity and *L. crispatus* associated with menopause. Seeing that the study that most closely reflected our findings was a meta-genomic analysis of 516 women to evaluate the effect of lifestyle on the cervical microbiome (Jie et al. 2021), we find confirmation on the one hand and point out on the other hand that microbiome-based studies need to be conducted on large sample sizes. Moreover, just as in mentioned study, we find a significantly larger prevalence of *L. crispatus* among premenopausal women on oral contraceptives and no linkage between *G. vaginalis* and a disturbed microenvironment. In the same study, *L. vaginalis* was positively correlated with irregular menstruation, while in ours, with increasing PAP-score.

Regarding the role of *U. parvum* in the progression of neoplasia, we found three reports in the literature on the possible association between *U. parvum*, HPV, and intraepithelial neoplasia of the cervix (Biernat-Sudolska et al. 2011; Szostek et al. 2014; Drago et al. 2016), indicating the need for further investigation, as the detection of *U. parvum* is currently not indicative for therapy (Patel and Nyirjesy 2010; Kokkayil and Dhawan 2015).

In our study, only a few additional CIN III cases were identified. Results from other centers must be awaited to determine whether the addition of HPV testing will improve cervical cancer screening in Germany. In contrast to many other studies, dyplastic changes were only associated with *U. parvum*. We believe that there is currently insufficient data to support modulation of the vaginal microbiome, which is currently heavily marketed to counter dysplastic changes.

## Supplementary Information

Below is the link to the electronic supplementary material.

Supplementary file1 (PDF 88 KB)

## Data Availability

The datasets generated during the current study are available from the corresponding author on reasonable request.

## Code availability

Software packages are published, available to the public, and sources are cited in the Methods section.
