# Supplementary figures and images for: Analysis of the cervical microbiome in women from the German national cervical cancer screening program
Source: J Cancer Res Clin Oncol. 2023 Feb 13;149(9):6489–500. doi: 10.1007/s00432-023-04599-0 (PMC10356625; doi:10.1007/s00432-023-04599-0)

# Suppl. Figure A1

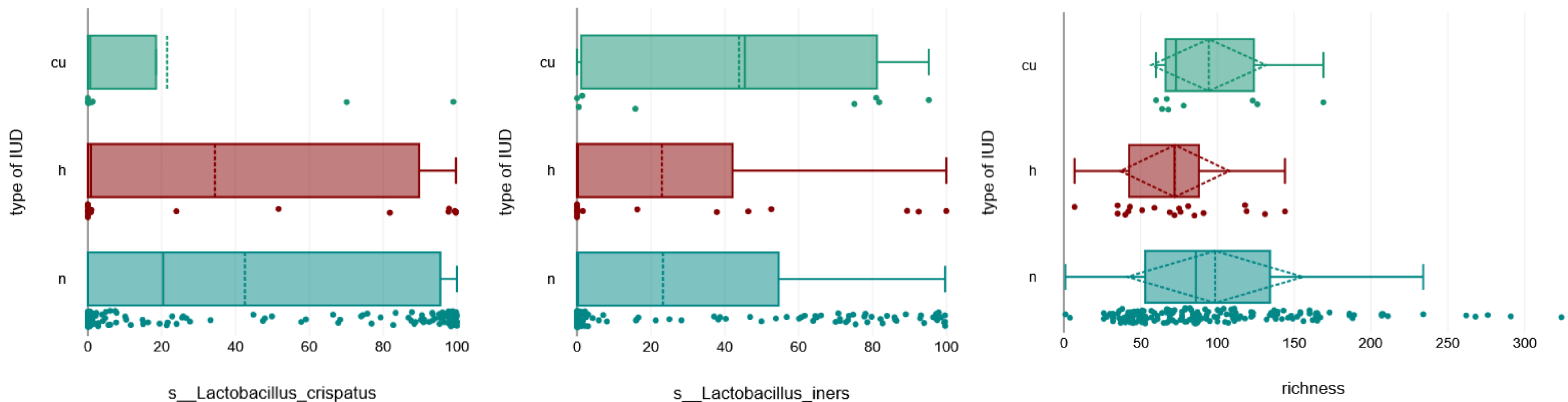

# Suppl. Figure A2

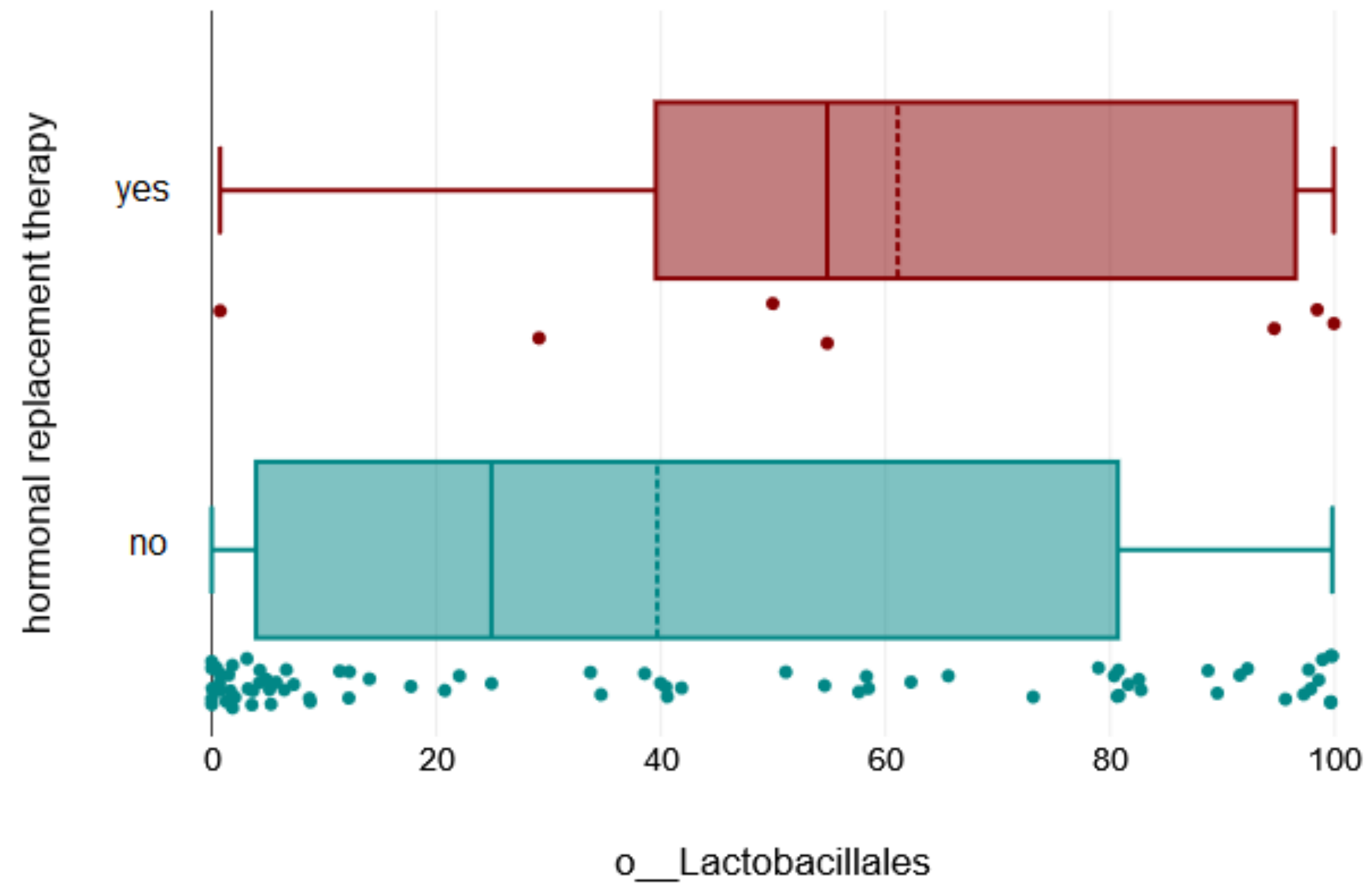

Supplement: Supplementary file 1 — Supplementary file1 (PDF 88 KB) [file 432_2023_4599_MOESM1_ESM.pdf]
